# Linking HIV-Infected TB Patients to Cotrimoxazole Prophylaxis and Antiretroviral Treatment in India

**DOI:** 10.1371/journal.pone.0005999

**Published:** 2009-06-22

**Authors:** Neeraj Raizada, Lakbir Singh Chauhan, B. Sai Babu, Rahul Thakur, Ajay Khera, D. Fraser Wares, Suvanand Sahu, D. Bachani, B. B. Rewari, Puneet K. Dewan

**Affiliations:** 1 Formerly of the Office of the WHO Representative to India, New Delhi, India; 2 Central Tuberculosis Division, Directorate General of Health Services, Ministry of Health and Family Welfare, New Delhi, India; 3 Department of Health, State Government of Andhra Pradesh, Hyderabad, India; 4 Office of the WHO Representative to India, New Delhi, India; 5 National AIDS Control Organization, Ministry of Health and Family Welfare, New Delhi, India; 6 World Health Organization, Southeast Asia Regional Office, New Delhi, India; MRC Laboratories, Gambia

## Abstract

**Background:**

HIV-infected persons suffering from tuberculosis experience high mortality. No programmatic studies from India have documented the delivery of mortality-reducing interventions, such as cotrimoxazole prophylactic treatment (CPT) and antiretroviral treatment (ART). To guide TB-HIV policy in India we studied the effectiveness of delivering CPT and ART to HIV-infected persons treated for tuberculosis in three districts in Andhra Pradesh, India, and evaluated factors associated with death.

**Methods and Findings:**

We retrospectively abstracted data for all HIV-infected tuberculosis patients diagnosed from March 2007 through August 2007 using standard treatment outcome definitions. 734 HIV-infected tuberculosis patients were identified; 493 (67%) were males and 569 (80%) were between the ages of 24–44 years. 710 (97%) initiated CPT, and 351 (50%) collected >60% of their monthly cotrimoxazole pouches provided throughout TB treatment. Access to ART was documented in 380 (51%) patients. Overall 130 (17%) patients died during TB treatment. Patients receiving ART were less likely to die (adjusted hazard ratio [HR] 0.4, 95% confidence interval [CI] 0.3–0.6), while males and those with pulmonary TB were more likely to die (HR 1.7, 95% CI 1.1–2.7, and HR 1.9, 95% CI 1.1–3.2 respectively).

**Conclusions:**

Among HIV-infected TB patients in India death was common despite the availability of free cotrimoxazole locally and ART from referral centres. Death was strongly associated with the absence of ART during TB treatment. To minimize death, programmes should promote high levels of ART uptake and closely monitor progress in implementation.

## Introduction

India has the world's highest burden of tuberculosis (TB) with 1.9 million estimated incident cases per year. It also ranks among the world's highest HIV burden with an estimated 2.3 million persons living with HIV/AIDS [1], [2]. Tuberculosis is much more likely to be a fatal disease among HIV-infected persons than persons without HIV infection [3], [4], [5]. The World Health Organization (WHO) guidelines for the management of HIV-infected persons with tuberculosis recommend the provision of cotrimoxazole prophylactic treatment (CPT) and, if indicated, antiretroviral treatment (ART) [6].

Cotrimoxazole is a broad-spectrum antimicrobial agent that is recommended as primary prevention against opportunistic infections in HIV-infected persons [7]. At the dose of sulfamethoxazole 800 mg/trimethoprim 160 mg daily, cotrimoxazole has been demonstrated to be effective at reducing mortality due to *Pneumocystis* pneumonia, toxoplasmosis, and other bacterial opportunistic infections [8], [9], [10]. In randomized studies, cotrimoxazole has reduced mortality in HIV-infected adults newly diagnosed with tuberculosis; however, data from programmes in resource-limited countries are scarce [11], [12], [13], [14]. In India, primary prevention with cotrimoxazole has been recommended by the National AIDS Control Programme (NACP) for all HIV-infected patients with WHO Clinical Stage III and IV or those in Stage II and with CD4 counts of <200 [15]. CPT prescription and delivery, however, has previously been left to the discretion of individual providers and has not been available at primary health centres where patients routinely access care.

Antiretroviral treatment has been convincingly associated with improved treatment outcomes and reduced mortality [9], [16], [17], [18]. India has made free antiretroviral treatment widely available nationwide through a growing treatment network that, as of June 2008, has initiated 134,927 persons on ART at 157 ART centres [19]. HIV-infected persons are eligible for ART after counseling, clinical examination, and if immunologic criteria are met; for those with tuberculosis, CD4 cell count <350/ml indicates ART eligibility [20]. No data regarding the effectiveness of referral to the ART centre network has been reported, and systematic referral of HIV-infected TB patients for ART was previously not practiced.

To guide TB-HIV policy in India, the Government of India's Revised National TB Control Programme (RNTCP) and NACP implemented a demonstration project in three districts in the state of Andhra Pradesh. The project's goal was to deliver the WHO-recommended interventions of CPT and ART (as per national guidelines) to all known HIV-infected TB patients. We retrospectively evaluated the efficiency of decentralized CPT delivery from primary health centres, assessed the effectiveness of referral links to ART centres, and studied factors associated with mortality among HIV-infected individuals treated for tuberculosis under programmatic conditions.

## Methods

### Setting

Three districts in the southern Indian state of Andhra Pradesh—Ananthapur, Vizianagaram, and Vishakapatnam (combined 2001 population 9.7 million)—were intentionally selected to implement these TB-HIV activities on the basis of high HIV prevalence among antenatal clinic attendees during NACP surveillance from 2003–2006. In the three districts there are a total of 154 primary health care facilities which provide general health services including TB diagnostic and treatment services. At the time of implementation of these interventions, there were 47 HIV counseling and testing centres and 3 ART centres for free HIV diagnosis and treatment (2 ART centres in Ananthapur, 1 in Vishakapatnam and none in Vizianagaram). HIV treatment and care, including ART, was available free of cost to patients at these ART centres. Patients from Vizianagaram district were able to access ART services in the adjacent district of Vishakapatnam.

### Interventions

TB diagnosis under RNTCP is based on initial smear microscopy, but allows for the diagnosis of smear-negative and extrapulmonary TB as well. Pulmonary TB diagnosis is based on a standard diagnostic algorithm [21]. Patients identified as TB suspects are subject to sputum microscopy; those with sputum microscopy results positive for acid-fast bacilli are declared TB cases, and patients with negative sputum results are offered a course of antibiotics. Patients who remain symptomatic after antibiotics are offered repeat sputum examination and, if still sputum-negative, a chest radiograph. On the basis of negative sputum results and clinical and radiographic examination, any clinician can declare any patient to be a case of smear-negative pulmonary TB. Extrapulmonary TB is generally left to the provider to declare diagnosis on clinical and pathological examination, but standard algorithms are provided for lymph node and pleural tuberculosis. All TB patients received free, standardized anti-TB treatment as per national guidelines [21]. For new patients RNTCP uses an intensive phase regimen of isoniazid, pyrazinamide, rifampicin, and ethambutol dosed thrice-weekly for 2 months under direct observation, and a continuation phase regimen of isoniazid and rifampicin dosed thrice-weekly for 4 months with direct observation of at least the first dose per week. For re-treatment patients, RNTCP uses an intensive phase of isoniazid, pyrazinamide, rifampicin, and ethambutol dosed thrice weekly for 3 months (along with streptomycin for the first 2 months), and a continuation phase regimen of isoniazid, rifampicin, and ethambutol dosed thrice-weekly for 6 months. In any regimen, those smear-positive patients who remain smear-positive after the normal intensive phase treatment have their intensive phase extended by one month. Treatment duration is determined by the number of doses, and the durations above assume that all doses are taken; missed doses lead to a longer total duration of treatment.

Providers were instructed to immediately refer all TB patients with any HIV risk factors to the nearest HIV counseling and testing centre, as per the prevailing national guidelines at the time. HIV test results were communicated to the referring provider either directly by the patient or via direct communication from HIV counselor to the referring provider (with patient assent). An initial 1-month supply of CPT, at the dose of sulfamethoxazole 800 mg/trimethoprim 160 mg daily (one “double strength” cotrimoxazole tablet) was provided to the HIV-infected TB patients from their respective primary health centres, and 1-month refills made available for the duration of their TB treatment at no cost. While being providing CPT, patients were counseled on the benefits of CPT, possible side effects, and the importance of adherence. In an attempt to address concerns about patient privacy, CPT was available at primary health centres only from health care workers, and not through community DOT providers. At the start of the intervention, CPT became available for all HIV-infected TB patients at the same time, regardless of whether a patient was newly diagnosed with TB or already on TB treatment for several months.

Providers were asked to refer HIV-infected TB patients to the nearest ART centre as soon as possible, but at least 2 weeks after the initiation of anti-TB treatment (to address the infection control concerns). Patients were asked to continue CPT from ART centres after their TB treatment was completed, or earlier if ART were initiated during TB treatment.

The RNTCP individual patient TB treatment cards were modified to enable recording of HIV status, CPT initiation, CPT adherence, and ART information. Trainings were conducted for all district medical officers, programme staff of NACP and RNTCP, and pharmacists at peripheral health facilities. Standard forms were developed to facilitate referrals and feedback on results of HIV testing and ART evaluations. TB programme staff maintained a separate register of HIV-infected TB patients to facilitate supervision and monitoring.

### Definitions

Standard definitions were used to categorize patients according to TB type and treatment outcomes [21]. New cases were defined as those TB patients who had less than 1-month of previous anti-TB treatment. Treatment success was defined as having a treatment outcome of cured or completed treatment as documented on the TB treatment card. Any death that occurred during TB treatment was classified as “death during treatment”. If the date of ART or CPT treatment initiation was more than 1 week before TB treatment initiation, that person was said to be on ART or CPT prior to TB treatment. Patients who initiated ART at any time before TB treatment was completed were classified as having received ART during TB treatment.

### Data collection and analysis

We retrospectively abstracted data from programme records for all HIV-infected TB patients notified to the RNTCP between 1 March and 31 August 2007. Treatment cards were collected from all health facilities and data double entered with Microsoft Excel. At the end of 6 months RNTCP and NACP conducted field visits to 30 health service delivery sites in the three districts, and conducted unstructured interviews with 100 randomly selected HIV positive TB patients to identify gaps in the delivery of intervention services.

We compared HIV-infected TB patients who died during TB treatment with those who did not die. Risk factors for death analyzed included age, sex, district of residence, type of TB, CPT use, CPT adherence, and ART provision. To assess CPT adherence in the context of a variable duration of TB treatment, we expressed the number of monthly CPT dose pouches picked up—as recorded on TB treatment cards—as a proportion of the number of months which an HIV-infected TB patient was alive and on treatment (rounded down to the nearest month). Data on CD4 cell counts were not available in routine records.

For bivariate analysis, we compared proportions using chi-square, and for continuous variables we compared medians using the Wilcoxin Rank-Sum test. The survival distribution of the overall cohort was estimated using the Kaplan-Meier method, and subgroups were compared using the Cox-Mantel (log-rank) test. P-values throughout were 2-sided and we used a 0.05 significance level.

For multivariate analysis we calculated hazard ratios for factors associated with death using a Cox proportional-hazards model. The dependent variable was the number of days. Treatment outcomes other than death were censored after the last day of TB treatment recorded. We pre-selected variables for inclusion in the multivariable model based on prior information from studies from other settings on risk factors for mortality in HIV-infected TB patients [9], [11], [17], [18], [22]. The analysis was conducted using EpiData 2.0 (EpiData Association, Odense Denmark) and SPSS 14.0 (SPSS Inc., Chicago, USA).

### Ethical issues

This retrospective analysis was conducted after review and approval by the National AIDS Control Programme and the Central TB Division, Directorate General of Health Services, Ministry of Health and Family Welfare, Government of India. The activity was determined to be programme evaluation of the implementation of internationally-recommended interventions. Electronic databases created for this analysis were stripped of personal health identifiers and maintained securely.

## Results

Among the 7,752 patients registered in the three districts from March 1 2007 to August 31 2007, 734 (9.5%) were identified as HIV-infected. The proportion of registered patients reported to be HIV-infected varied by district; 322 (9.9%) out of the 3,239 of TB patients registered from Ananthapur were HIV-infected, as were 317 (11.6%) out of the 2,725 from Vishakapatnam and 95 (5.3%) out of the 1,788 from Vizianagaram. Characteristics of these 734 HIV-infected TB patients are shown in **Table 1**. Of these, 493 (67%) were male and the median age was 34 years (range 8–89 years); 80% were between the ages of 25 and 46 years.

**Table 1:** Characteristics of HIV-infected TB patients, Andhra Pradesh, India, 2007 (N = 734).

Characteristic	Subcategory	N (%)
District of Residence	Ananthapur	322 (43.8)
	Vishakapatnam	317 (43.2)
	Vizianagaram	95 (13.0)
Sex	Male	493 (67.2)
	Female	220 (30.0)
	Not available	21 (2.8)
Previous TB treatment	Yes	99 (13.5)
	No	635 (86.5)
Type of TB	Pulmonary	551 (75.1)
	Extrapulmonary	183 (24.9)
TB Classification	New smear-positive pulmonary	277 (37.7)
	New smear-negative pulmonary	190 (25.9)
	New extrapulmonary	168 (22.9)
	Re-treatment	99 (13.5)
Age Group	<15 years	1 (0.1)
	15–24 years	50 (6.8)
	25–34 years	318 (43.3)
	35–44 years	251 (34.2)
	45–54 years	84 (11.4)
	>55 years	27 (3.7)
	Not available	3 (0.4)
On ART before TB treatment	No	601 (81.9)
	Yes	133 (18.1)
On ART at any time during TB treatment	No	354 (48.2)
	Yes	380 (51.8)

Abbreviations: TB–Tuberculosis; ART–antiretroviral treatment.

### Access to CPT

710 (97%) of the 734 known HIV-infected TB patients were initiated on CPT during the evaluation period; 22 (3%) of patients were already taking CPT before the start of TB treatment from the ART centre, and no record of CPT initiation was found for two patients. Nearly half (323, 44%) received prophylaxis within 1 week of starting TB treatment **(** **Table 2** **)**. The exact date of CPT initiation was not available for 26% of patients, as field staff commonly only recorded the month of initiation. Overall 351 (48%) collected ≥60% of the number of monthly cotrimoxazole pouches provided. Anecdotally, we observed that the on-site availability of CPT at primary health centers appeared to motivate providers assess the HIV status of TB patients.

**Table 2:** CPT initiation delay and adherence among HIV-infected TB patients, Andhra Pradesh 2007 (N = 734).

Characteristic	Sub-category	N (%)	Cumulative N (%)
Delay of initiation of CPT, relative to date of TB treatment initiation	0 days (Before or on the same day)	203 (27.6)	203 (27.6)
	1–7 days	120 (16.4)	323 (44.0)
	8–30 days	131 (17.9)	454 (61.9)
	30–60 days	48 (6.5)	502 (68.4)
	61–120 days	16 (2.2)	518 (70.6)
	CPT started from ART center	22 (3.0)	540 (73.6)
	Data not available	194 (26.4)	734 (100)
Proportion of monthly CPT packets documented as picked up*	81–100%	214 (29.2)	214 (29.2)
	61–80%	137 (18.6)	351 (47.8)
	41–60%	88 (12.0)	439 (59.8)
	21–40%	109 (14.9)	548 (74.5)
	0–20%	111 (15.1)	659 (89.7)
	Not available	75 (10.2)	734 (100)

* Relative to total duration of TB treatment taken during the evaluation period.

Abbreviations: CPT–cotrimoxazole prophylactic treatment; TB–tuberculosis; ART–antiretroviral treatment.

### Access to ART

Among the 734 HIV-infected TB patients, 133 (18%) were documented as already on ART at the time of TB treatment initiation. Of the 601 HIV-infected TB patients not on ART at the time of TB treatment initiation, 559 (93%) were documented to have been referred to ART centres. Of the 559 referrals to ART centre, 324 (61%) were documented to be subsequently pre-registered at ART centres. Of the 324, 247 (76%) were initiated on ART by the end of TB treatment. In total, 380 of 734 (52%) had access to ART during TB treatment.

### TB treatment outcomes

Treatment outcomes were available for all 734 HIV-infected TB patients **(** **Table 3** **)**. Treatment success was reported in 536 (73%) patients; treatment success was highest among new-extrapulmonary patients (89%), and was lowest among re-treatment patients (51%). Treatment default was more common among re-treatment patients (16%) or new smear-negative TB patients (11%), compared to new smear-positive TB patients (4.3%) (p<0.01 for each comparison). The combined treatment success rates in this group of HIV patients was lower than the overall treatment success rate in the three districts (84%) during the same time period.

**Table 3:** Tuberculosis treatment outcomes among HIV-infected TB patients, Andhra Pradesh 2007 (N = 734).

Treatment outcome	NSP (%)	NSN (%)	NEP (%)	Re-treatment (%)	Total (%)
Treatment success	201 (72.4)	136 (71.6)	149 (88.7)	50 (50.5)	536 (73.0)
Default	12(4.3)	20 (10.5)	7 (4.2)	16 (16.2)	55 (7.5)
Failure	9(3.3)	1 (0.5)	0	3 (3.0)	13 (1.8)
Died	55 (19.9)	33 (17.4)	12 (7.2)	30 (30.3)	130 (17.7)
Total	277 (100)	190 (100)	168 (100)	99 (100)	734 (100)

Abbreviations: NSP–new pulmonary tuberculosis with sputum smears positive for acid-fast bacilli; NSN–new pulmonary tuberculosis with sputum smears negative for acid-fast bacilli; NEP–New extrapulmonary tuberculosis.

### Risk factors for death during TB treatment

**Figure 1:**
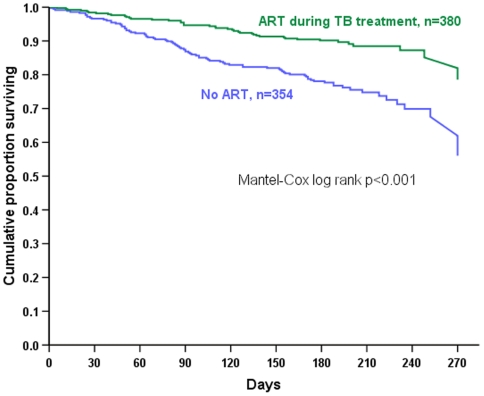
Kaplan-Meier survival curve for HIV-infected TB patients, by exposure to antiretroviral treatment. ART = antiretroviral treatment, TB = tuberculosis.

Overall 130 (18%) HIV-infected TB patients died during TB treatment. Bivariate risk factors for death are shown in **Table 4**. HIV-infected female patient had a lower risk of death than males (relative risk [RR] 0.55, 95% CI 0.37–0.83), and re-treatment patients were more likely to die than new patients (RR 1.92, 95% CI 1.36–2.73). Death occurred in 43 of 380 (11.3%) patients exposed to ART during TB treatment, compared with 87 of 354 patients (24.6%) without ART (RR 0.46, 95% CI 0.33–0.64). Kaplan Meier plots showed that divergence in survival between those with and without ART exposure appeared after approximately 2 months of anti-TB treatment **(** **Figure 1** **)**.

**Table 4:** Bivariate analysis of risk factors for mortality during TB treatment among HIV-infected TB patients, Andhra Pradesh 2007 (N = 734).

Characteristic	Sub-Category	Total	Died, N (%)	RR (95% CI)
District of Residence	Ananthapur	322	56 (17.4)	Referent
	Vishakapatnam	317	60 (18.9)	1.09 (0.78–1.51)
	Vizianagaram	95	14 (14.7)	0.85 (0.49–1.45)
Sex	Male	493	102	Referent
	Female	220	25 (11.4)	0.55 (0.37–0.83)
	Not available	21	3 (14.9)	0.69 (0.24–2.00)
Previous TB treatment	No	635	100 (15.8)	Referent
	Yes	99	30 (30.3)	1.92 (1.36–2.73)
Type of TB	Pulmonary	551	113 (20.5)	Referent
	Extrapulmonary	183	17 (9.3)	0.45 (0.28–0.73)
Tuberculosis Classification	New Smear-Positive	277	55 (19.9)	Referent
	New Smear-Negative	190	33 (17.4)	0.87 (0.59–1.29)
	New Extrapulmonary	168	12 (7.1)	0.36 (0.20–0.65)
	Re-Treatment (all types)	99	30 (30.3)	1.53 (1.04–2.23)
Age Group	<15 years	1	0	
	15–24 years	50	5	0.59 (0.25–1.04)
	25–34 years	318	54	Referent
	35–44 years	251	46	1.08 (0.76–1.54)
	45–54 years	84	17	1.19 (0.73–1.94)
	>55 years	27	8	1.74 (0.93–3.28)
	Data not available	3	0	
Delayed CPT initiation	<1 week	323	58 (18.0)	Referent
	1 week–2 months	179	30 (16.7)	0.93 (0.62–1.39)
	>2 months	16	1 (6.3)	0.35 (0.05–2.4)
	Data not available	194	36 (18.6)	1.03 (0.71–1.51)
CPT adherence	>80%	214	39 (18.2)	Referent
	60–80%	137	30 (21.9)	1.20 (0.79–1.84)
	40–60%	88	13 (14.8)	0.81 (0.46–1.44)
	<40%	220	28 (12.7)	0.70 (0.45–1.09)
	Data not available	75	20 (26.7)	1.46 (0.91–2.34)
ART during TB treatment	No	354	87 (24.6)	Referent
	Yes	380	43 (11.3)	0.46 (0.33–0.64)

In multivariate analysis **(** **Table 5** **)**, ART exposure at any time during TB treatment remained independently protective against death (adjusted hazard ratio [HR] 0.41, 95% CI 0.28–0.60). Being a male and having pulmonary TB were also independently associated with a greater risk of death, and there was a trend towards greater risk of death in those with prior anti-TB treatment.

**Table 5:** Multivariate analysis of risk factors for mortality during TB treatment among HIV-infected TB patients, Andhra Pradesh 2007 (n = 713).

Characteristic	Adjusted Hazard Ratio	95% Confidence Interval
Antiretroviral treatment during TB treatment	0.41	0.28–0.60
Male sex	1.67	1.07–2.62
Previous tuberculosis treatment	1.44	0.93–2.23
Pulmonary tuberculosis	1.89	1.11–3.21
Age Group		
15–24 years	0.75	0.30–1.90
25–34 years	Referent	
35–44 years	0.89	0.61–1.34
45–54 years	1.12	0.68–2.00
>55 years	1.98	0.94–4.19

Limiting the multivariate analysis to those patients who started ART during TB treatment ( i.e. excluding the 133 who were already on ART at the time of TB diagnosis) did not substantially change the association between ART exposure and lower risk of death (HR 0.32, 95% CI 0.20–0.52), nor did limiting the analysis to males or females alone (HR 0.47, 95% CI 0.32–0.71 and HR 0.23, 95% CI 0.08–0.64).

## Discussion

This evaluation provides the first evidence from India regarding the operational challenges of delivering WHO-recommended mortality-reducing TB-HIV interventions under general field conditions. It also provides information on the daunting problem of the high mortality of HIV-infected TB patients. In this resource-limited setting, nearly 1 in 5 HIV-infected individuals diagnosed with TB died during TB treatment. While previous studies have reported high mortality among HIV-infected persons with tuberculosis [17], [18], [23], the high death rate we observed in Andhra Pradesh was particularly concerning given the provision of free CPT and reasonable levels of ART uptake. These findings imply that very high levels of ART uptake may be required to substantively reduce tuberculosis case fatality rates.

With no additional resources or efforts beyond provider trainings and monitoring by regular HIV and TB programme staff, more than 95% of all detected HIV-infected TB patients were initiated on CPT. Mixed adherence may have reduced the effectiveness of CPT in this population, as just half of patients collected >60% of their cotrimoxazole. With increased awareness of CPT, adherence may improve; additional measures the programmes should consider include use of peer-counselor networks and non-governmental organizations to support and encourage patients and providers.

We found that patients provided ART during TB treatment had less than half the risk of death as those not provided ART. This finding was independent of sex or previous TB treatment history, and is consistent with findings from several other settings [17], [18], [24], [25]. This evidence from India strongly reinforces the interpretation that ART is the single most potent intervention available for public health programmes to reduce death during TB treatment among HIV-infected individuals. Regardless of CPT availability, in this setting the linkages to ART were arguably inefficient. Of the 600 HIV-infected TB patients not already on ART, despite evidence of referral, only 229 (38%) started ART during TB treatment. Even when we included patients already on ART before TB treatment, in total only 50% of TB patients received any ART during TB treatment. While all of the remaining patients may not have been immediately eligible for ART, given the strong protection against death during TB treatment, the TB and HIV programmes must urgently seek improvements in ART referral efficiency and treatment uptake.

HIV-infected males experienced higher death rates than females; the reasons for this finding are unclear. Overall case-fatality rates among males with tuberculosis in India are modestly higher than in females, and postulated reasons for higher mortality among males include differences in treatment adherence, age distribution, smoking behavior, and comorbidities [26], [27], [28]. Regardless, ART was associated with similar protection from mortality in males and females alike. The 104 patients registered as retreatment cases suffered from poor treatment success (50%) and high case-fatality (30%). No information was available regarding patient co-morbidities or drug resistance, but the very high death rates among previously-treated patients urgently warrants further exploration.

### Limitations

Our evaluation was subject to several important limitations. This was a retrospective evaluation of a demonstration conducted under routine programme conditions; available information was limited to that recorded on standard patient records which had been modified to include additional HIV-related information. With ascertainment of HIV-infection among TB patients based on selective referral of those persons with HIV risk factors, the patients identified might not be representative of the larger population of HIV-infected TB patients. All 3 districts practiced the same selective referral policy, but in Vizianagaram district a simultaneous population-based survey of HIV infection among TB patients provides insight into the effectiveness of the ascertainment of HIV infection [29]. In Vizianagaram, the survey estimated a 6.5% (95% CI 4.5–9.4%) prevalence of HIV infection in TB patients, which would imply that there were approximately 116 (81–168) HIV-infected TB patients among the cohort of 1788 registered from Vizianagaram in this study period. This evaluation documented 95 HIV-infected TB patients from Vizianagaram, or 82% of the expected number, suggesting that the ascertainment as sufficiently efficient to expect that these results could be generalized to all HIV-infected TB patients in these districts.

Treatment outcomes were recorded by many providers, and were not independently validated. Information about other biomedical risk factors for death that may have confounded this evaluation, such as severity of TB disease, drug resistance, severity of immune suppression, or comorbidities, were not available. No ‘control’ areas without CPT were assessed because we did not seek to evaluate the efficacy of CPT as an intervention, and observational analysis of the association of CPT and mortality were not possible since almost all patients were exposed to CPT during TB treatment. The efficacy of CPT, however, has been amply demonstrated in multiple settings. The recording of ART information was dependent on feedback from the ART centre. Some patients may not have had ART consumption recorded on their TB treatment card, so we may have misclassified these patients as not provided ART, and thus underestimated the protective effect of ART in this population. We were also unable to collect adequate information about sub-type of extrapulmonary TB, patient CD4 count, or the specific timing of ART initiation. Future studies should seek to collect this information to better understand the finding of lower overall mortality in extrapulmonary TB, and the protective effect of ART against mortality during TB treatment.

### Translating findings into policy and practice

On the basis of this experience, decentralized CPT delivery for HIV-infected TB patients has been included in national TB-HIV policies, and is being presently implemented in high HIV-prevalence settings throughout India [30]. Joint TB-HIV trainings now strongly emphasize the referral of HIV-infected TB patients to ART centres. TB patient records and RNTCP registers have been modified to include information on HIV status, CPT, and ART. These recording enhancements will allow for routine programme monitoring and supervision of the local programme efficiency in detecting HIV-infection among TB patients and linking these patients to CPT and ART. Operational research is ongoing in other districts in India to understand weaknesses in referral to ART centres and ART uptake.

### Conclusions

Cotrimoxazole prophylaxis can be delivered to HIV-infected TB patients under programmatic conditions in India. Despite the availability of free cotrimoxazole locally and ART from referral centres, death was common and was strongly associated with the absence of ART during TB treatment. These findings have been translated into national policy and programme implementation through the expansion of cotrimoxazole availability and training on the importance of ART. To minimize death, the TB and HIV programmes should target high levels of ART uptake and closely monitor progress in implementation.
